# Supplementary material for: Medication-Related Complaints in Residential Aged Care
Source: Pharmacy (Basel). 2023 Mar 23;11(2):63. doi: 10.3390/pharmacy11020063 (PMC10142598; doi:10.3390/pharmacy11020063)
Supplement: Supplementary file 1 [file pharmacy-11-00063-s001.zip › pharmacy-2220057-supplementary figure.pdf]

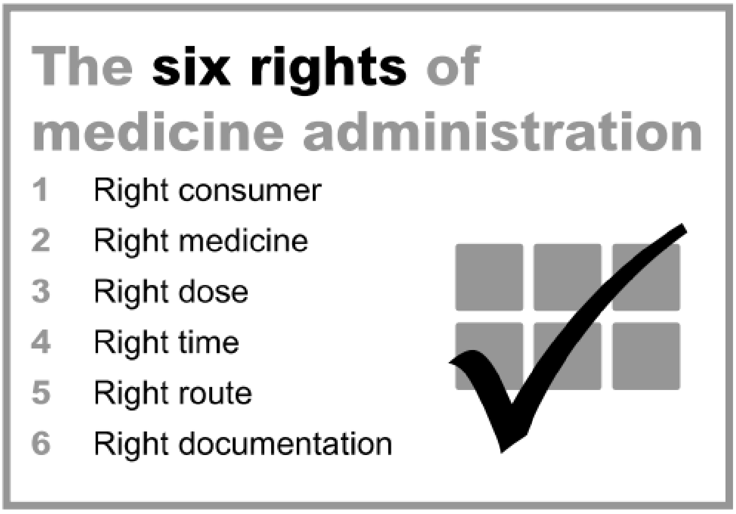

Supplementary Figure S1. Six rights of medication administration [19].
