# Supplementary material for: Medication-Related Complaints in Residential Aged Care
Source: Pharmacy (Basel). 2023 Mar 23;11(2):63. doi: 10.3390/pharmacy11020063 (PMC10142598; doi:10.3390/pharmacy11020063)
Supplement: Supplementary file 1 [file pharmacy-11-00063-s001.zip › pharmacy-2220057-supplementary tables.pdf]

Supplementary Table S1: Questions asked when lodging a Complaint

No.	Question
1	What type of compliant would you like to submit? (open/confidential/anonymous)
2	Have you signed the notice of collection which explains how the Commission will use personal information collected?
3	What is your name and address? (only if an open or confidential complaint)
4	Do you want us to call or email you about your complaint? (only if an open or confidential complaint)
5	My complaint relates to; a) the care or services I am receiving; or b) the care or services someone else is receiving.
6	Details of person receiving care (only if an open or confidential complaint)
7	Address of person receiving care (only if an open or confidential complaint)
8	If you are lodging this complaint on behalf of someone else what is your relationship with the person receiving care?
9	If you are lodging this complaint on behalf of someone else is the person receiving care aware of this complaint?
10	If you are lodging this complaint on behalf of someone else, are you authorised to make decisions on behalf of the person receiving care?
11	What type of aged care service does the complaint or information relate to?<br>a. residential aged care b. home care
12	Name of service?
13	What does your complaint relate to?<br>a. Abuse b. Choice and Dignity c. Client Assessment and Service d. Consultation and Communication e. COVID f. Financial g. Food and Catering h. Goods and Equipment i. Health Care j. Infection control k. Personal Care l. Personal Property m. Physical Environment n. Respite o. Social and Domestic Assistance p. Termination of agreement or q. Other.
14	Tell us more details about your complaint?
15	If available, upload supporting information about your complaint
16	Have you raised your complaint directly with the service or someone else?
17	Would you like to be involved in the resolution process? (only if an open or confidential complaint)
18	Would you like feedback on the resolution of this complaint? (only if an open or confidential complaint)
19	Would you like the service provider to contact you directly about your concerns? (only if an open or confidential complaint)
20	What outcome would you like from this complaint?

Supplementary Table S2. NCCIMS keywords and sub-keywords included in dataset extract

Keyword	Sub-Keyword
Abuse	Physical
	Psychological/emotional
Choice and Dignity	Right to refuse medication
Client assessment/Service implementation	Polypharmacy review
Consultation and communication	Ability to express needs/wants
	Information about medication
Goods and equipment	Medical and pharmaceutical supplies
Health Care	Allied health assessment and services
	Chemical restraint
	Constipation and continence management
	Dementia management
	Falls prevention and post falls management
	Infectious diseases and infection control
	Medication administration and management
	Pain management
	Palliative/end of life care
	Physical restraint
	Behaviour management
	Mental health
Personal care	Personal safety & interventions
	Sleep
